# Assessment of female breast dose for thoracic cone-beam CT using MOSFET dosimeters

**DOI:** 10.18632/oncotarget.15555

**Published:** 2017-02-21

**Authors:** Wenzhao Sun, Bin Wang, Bo Qiu, Jian Liang, Weihao Xie, Xiaowu Deng, Zhenyu Qi

**Affiliations:** ^1^ Department of Radiation Oncology, Sun Yat-Sen University Cancer Center, Collaborative Innovation Center for Cancer Medicine, State Key Laboratory of Oncology in South China, Guangzhou 510060, China

**Keywords:** radiotherapy, cone beam computed tomography (CBCT), metal oxide semiconductor field-effect transistor (MOSFET), breast dose, image registration

## Abstract

Objective: To assess the breast dose during a routine thoracic cone-beam CT (CBCT) check with the efforts to explore the possible dose reduction strategy.

Materials and Methods: Metal oxide semiconductor field-effect transistor (MOSFET) dosimeters were used to measure breast surface doses during a thorax kV CBCT scan in an anthropomorphic phantom. Breast doses for different scanning protocols and breast sizes were compared. Dose reduction was attempted by using partial arc CBCT scan with bowtie filter. The impact of this dose reduction strategy on image registration accuracy was investigated.

Results: The average breast surface doses were 20.02 mGy and 11.65 mGy for thoracic CBCT without filtration and with filtration, respectively. This indicates a dose reduction of 41.8% by use of bowtie filter. It was found 220° partial arc scanning significantly reduced the dose to contralateral breast (44.4% lower than ipsilateral breast), while the image registration accuracy was not compromised.

Conclusions: Breast dose reduction can be achieved by using ipsilateral 220° partial arc scan with bowtie filter. This strategy also provides sufficient image quality for thorax image registration in daily patient positioning verification.

## BACKGROUND

The therapeutic goal of advanced radiotherapy techniques like intensity modulated radiation therapy (IMRT) has focused on the precise irradiation of target volume while sparing normal tissues and organs as much as possible. This has created the need for more accurate patient positioning, especially for the treatment of thoracic cancer. Various image-guided devices like on-board kilovoltage cone beam computed tomography (CBCT) have thus been increasingly implemented in IMRT practice [1, 2]. By taking CBCT images on a daily or weekly basis, the possible patient's setup error can be detected and subsequently adjusted prior to the treatment [3, 4]. Several pilot studies also explore the feasibility of fast dose calculation based on CBCT images, so as to perform adaptive radiotherapy to further improve the treatment geometrical accuracy [5–7]. The rapidly growing use of CBCT, however, raises the issue of additional imaging dose and concomitant increases in secondary cancer risk [8–10]. There is a general consensus that low dose protocols should be preferred whenever possible to reduce the imaging dose and volume of exposed anatomy [11]. This is particularly true for female breast during a thoracic CBCT for lung cancer, which is often inadvertently irradiated even though it is not the imaged tissue of interest.

Several investigations have revealed the absorbed breast dose from single kV-CBCT ranges from several mGy to a few tens of mGy, depending on different imaging equipments and scanning protocols [11–13]. The stochastic health risk as a result of thoracic CBCT is not trivial, considering that for every 0.1 Gy of low dose irradiation at the age of 20 in female patients, the life time attributable risk of developing breast cancer increases 0.4% [14]. In fact, the female breast is more radiosensitive than previously assumed, according to the newly modified tissue weighting factors. The International Commission on Radiological Protection (ICRP) has recently changed its value from 0.05 to 0.12 for the breast tissue [15].

To better estimate the health effects from imaging exposures, it is essential to determine individual organ doses associated with various imaging procedures during a radiotherapy episode. Conventionally, these point doses were often measured in an anthropomorphic phantom using LiF thermoluminescent dosimeters (TLD) [11, 13]. The major advantages of LiF TLDs are their small size and tissue equivalent response. But they are also known to be tedious to use. The fact that acquiring moderately accurate, reproducible results using TLDs requires a stringent pre-calibration, annealing and post-irradiation readout process hinders their use in the diagnostic radiology clinic. Recently, the utilization of metal oxide semiconductor field effect transistor (MOSFET) technology in CT dosimetry has begun to appear in many peer-reviewed radiology publications [16–19]. Compared with TLDs, the MOSFET dosimeters can offer a fast, simple and inexpensive means to conduct a point dose measurement in radiation beams.

While the CBCT dose to the female breast has been previously investigated [11–13], the possible dose reduction strategies are less reported. A newly published paper discussed one feasible way to reduce additional dose to the patient due to CBCT by decreasing the mAs per frame and the number of projections per CBCT and concluded that image registration can be successfully performed even for lowest possible settings [20]. However, this pilot study only explored the effect of total mAs on image registration and there maybe have some other solutions such as partial arc scanning. Also direct dose measurement may better assess the breast dose during CBCT than the CT dose index (CTDI) used. In this study, we will focus on the imaging exposure to the healthy breast in daily thoracic CBCT for lung cancer patient positioning. The absorbed dose to the breast was measured with MOSFET dosimeters in a female-configured anthropomorphic phantom. Different scanning protocols were selected and the effects on image quality were analyzed. Proper strategies have been developed to reduce exposure in CBCT without reducing the image information on the registration process.

## RESULTS

### Calibration of the MOSFET dosimeter

The derived MOSFET sensitivity was 15.77±0.14 mV/cGy and 14.88±0.11 mV/cGy for blank field and filtrated field, respectively (Table 1). The average reading of repeated measurements for the typical thoracic CBCT (protocol B, no filtration) was 27.23 mV±0.73 mV, resulting in a measurement reproducibility of 2.7% at the tested dose level.

**Table 1:** Results and parameters for MOSFET calibration

Peak Voltage (kV)	Scanning configuration	HVL^a^ (mm Al)	Effective Energy (keV)	(μ_en_/ρ)_w,air_	B_W_	P_stem_	Sensitivity (mV/cGy)
120	F0+ S20	8.1	56.5	1.043	1.502	1	15.77
120	F1+ S20	9.0	60.0	1.047	1.509	1	14.88

^a^ Half value layer.

### Female breast dose of kV CBCT by ART phantom

No significant dependence was found between the breast surface dose and the breast volume (Table 2). The average breast surface doses of various breast volumes were 20.02 mGy and 11.65 mGy for bank field and filtrated field, respectively, indicating a dose reduction of 41.8% by use of bowtie filter.

**Table 2:** Breast surface doses of different breast volumes for thoracic CBCT scans (A, B, C, and D corresponds to the breast volume of 200, 400, 600, and 1200 cc respectively)

Filtration	A (200 cc)	B (400 cc)	C (600 cc)	D (1200 cc)
F0	19.44±1.05	20.29±0.00	19.44±1.05	20.92±0.00
F1	12.17±0.00	12.17±0.00	11.33±0.00	10.91±0.00

Protocol B with or without filter was used. The breast dose was presented in the form of D_mean_±SD mGy.

The doses of bilateral breasts measured by MOSFET dosimeters were shown in Table 3. It was observed that partial arc scanning produced a relatively lower breast dose than full arc mode. Particularly, the use of ipsilateral 220° partial arc significantly reduced the contralateral breast doses by about 16% and 44.4% for bank field and filtrated field, respectively, compared to the ipsilateral breast doses.

**Table 3:** Comparison of radiation doses delivered to ipsilateral breast vs. contralateral breast for full arc and partial arc CBCT

Filtration	Protocol B		Protocol C	
	Ipsilateral breast	Contralateral breast	Ipsilateral breast	Contralateral breast
F0	19.44±1.05	19.44±1.05	17.97±0.40	15.43±1.43
F1	12.17±0.00	12.17±0.00	11.33±0.00	6.30±0.69

The breast doses were measured using ART phantom with 200 cc artificial breast. The measured dose was presented in the form of D_mean_±SD mGy.

### Evaluation of CBCT image quality

For S20 collimator setting, both uniformity and LCV were improved by using bowtie filter (Table 4). As for M20 collimator setting, the bowtie filter improved the LCV while no improvements were seen on image uniformity.

**Table 4:** Results of uniformity and LCV tests for thoracic CBCT images

	Full Arc (F0M20)	Full Arc (F1M20)	Full Arc (F0S20)	Full Arc (F1S20)
Non-uniformity(%)	2.38	5.16	2.77	1.09
LCV(%)	1.16	1.11	1.24	0.64

### Registration accuracy

As shown in Table 5, image registration can be successfully performed for both CBCT protocols. It was noted that the systematic setup and registration errors were eliminated from the registration results. The maximum difference in registration between two protocols was observed to be within 0.1 cm.

**Table 5:** Comparison of registration results between full arc CBCT and partial arc CBCT for thoracic cancer patient positioning verification

Nominal shifted distance (cm)	Protocol B (full arc)			Protocol C (partial arc)		
	LAT	LONG	VERT	LAT	LONG	VERT
Normal setup (0)	0.04	0.14	0.04	0.06	0.16	0.06
Anterior 1cm	0.03	−0.11	−1.05	0.03	−0.05	−1.07
Superior 1cm	−0.01	−0.96	0.00	−0.02	−0.97	−0.01
Left 1cm	−0.96	−0.04	0.00	−0.96	−0.12	0.00

The systematic setup and registration error was subtracted from the registration results.

## DISCUSSION

The MOSFET dosimeters have been recommended by many authors for diagnostic CT dosimetry [16–19]. As the reproducibility of MOSFET dosimeters was closely correlated with the applied dose [21], the CMRP MOSFETs were calibrated at the dose range of a normal thorax CBCT, yielding a measurement accuracy within 3%. The derived MOSFET sensitivity was 14.88 and 15.77 mV/cGy for measurement with and without bowtie filter, respectively. This indicated a minimal sensitivity variation within 6% for blank and filtrated beam qualities, which is consistent with the previous finding [22]. The possible reason is that the filtration changes the energy spectrum and the high atomic number material in the sensitive volume of the MOSFET (silicon oxide) tends to over respond to the low energy x-rays due to photoelectric absorption effects. In this study, calibration was carried out individually for blank field and filtrated field for the purpose of accuracy.

The entrance surface dose was often used as an indicator of patient's exposure in radiographic applications. This is due to the fact that the kV radiation dose is greatest at the skin surface as well as the surface dose assessment is practical feasible. As shown by our results, the breast surface doses derived from a thorax CBCT, measured with MOSFET dosimeters, were on average 20.02 mGy. Although the observed imaging dose is small compared with the radiotherapy dose, the potential risk for stochastic effects could not be neglected considering the total dose from all imaging sessions during intensive IGRT is considerable. Kan et al [11] estimated that patient position verification using kV CBCT (Varian, OBI) on a daily basis (35 fractions) could cause 1.5 to 2.0 Gy extra dose to some critical organs, which might increase the overall risk of the secondary cancer by 3% to 4%. Considering XVI usually produces larger imaging dose than OBI [13], more attention should be paid to justification for use of XVI system.

Several efforts to reduce patient's exposure during CBCT were reported [20, 23–25]. These investigations were performed by minimizing the total mAs [20, 23] and the use of bowtie filter [24, 25]. Besides these, partial arc CBCT has recently be demonstrated to be a good supplement to current dose reduction strategies for patient positioning verification of head & neck and pelvis [25]. In this study, our results approved partial arc scan could also be used for thoracic cancer patient positioning by selecting proper scanning parameters. It was seen the use of 220° partial arc scan effectively reduced the radiation to the female breasts, while the image registration accuracy was not compromised. The observed dose reduction by partial arc may be due to the fact that 220° partial arc applied less number of projections and thus decreasing the total mAs. More importantly, ipsilateral 220° partial arc setting can minimize radiation exposures to the contralateral breast. This may be particularly suitable for routine CBCT in some cases such as breast cancer, in which sparing the heathy breast is one of major concerns.

The use of bowtie filter has several advantages, including a lower skin dose, reduction of the image saturation of the kV detector panel, and elimination of the cupping artifacts across the FOV [26]. As shown by our results, the introduction of bowtie filter produced significantly lower dose to the breasts compared to the none filtrated field, which was in consistent with previously published studies [24, 25]. In addition, CBCT acquisition using bowtie filter and S20 collimator produce much better image quality in terms of image uniformity and LCV, compared to F0 and S20 combination. The fact that CBCT acquisition with bowtie filter and M20 collimator yield relatively larger non-uniformity than F0 and M20 combination (5.16% vs 2.38%) may result from the off-axial kV field used by M20 setup. Consequently, the bowtie filter has been recommended as a routine setting for thoracic CBCT by our institutional protocol.

## CONCLUSIONS

The breast dose due to thoracic CBCT can be significantly reduced by using ipsilateral 220° partial arc scan with bowtie filter. This strategy also provides sufficient image quality for thorax image registration in daily patient positioning verification.

## MATERIALS AND METHODS

### kV CBCT

Experiments were performed on the X-ray Volume Imager (XVI) mounted onto an Elekta Synergy linear accelerator (Elekta, Crawley, UK). The XVI system can provide different combinations of kV collimators (labeled as 10 and 20 which can generate the axial field length of about 135mm and 143mm at the isocenter, respectively), field of views (FOV, labeled as S, M and L for small FOV (270 mm in diameter), medium FOV (410 mm in diameter) and large FOV (500 mm in diameter), respectively) and filtration cassettes (labeled as F0 and F1 for blank filter and bowtie filter, respectively). For a typical thorax CBCT scan, the medium FOV with collimator 20 (i.e. M20) was recommended by the manufacturer. However, the small FOV with collimator 20 (i.e. S20) was preferred in the calibration process as the central axis of the kV radiation is in line with the center of the kV detector panel in the small FOV configuration and offset in the M20 configuration. Also, the X-ray tube was stationed at 0° position rather than rotation during the calibration for the purpose of accuracy. The detailed scan parameters for MOSFET dosimeter calibration and thorax CBCT scan were given in Table 6. The acquired image data was processed by using the software XVI release 3.5 (Elekta, Crawley, UK).

**Table 6:** Scanning parameters for thorax CBCT. (Protocol A was used for MOSFET calibration, protocol B was used for nominal thorax CBCT scan, and protocol C was used for partial-arc scan investigation.)

	Protocol A	Protocol B	Protocol C
Tube voltage [kV]	120	120	120
Nonminal mA/frame	25	25	25
Nominal mS/frame	40	40	40
Frames	650	650	397
Total mAs	650	650	397
FOV [mm]	S	M	M
Collimator	20	20	20
Filter	F0/F1	F0/F1	F0/F1
Gantry Rotation	stationary x-ray tube (0°)	rotational x-ray tube (−180°~180°)	rotational x-ray tube (−110°~110°)

### MOSFET dosimetry system

The p-channel MOSFETs (brand named “MOSkin”) together with a portable reader were supplied by the Center of Medical Radiation Physics (CMRP), University of Wollongong, Australia. The physical performance of this dosimeter was well documented in the literature for external beam radiotherapy [27, 28], interventional radiology procedures [22] and diagnostic radiology dosimetry [16, 17].

The MOSFET dosimeter is connected to the CMRP reader system when it is used. The reader contains circuits not only for tracking and logging the threshold voltage but also for applying gate bias as required during exposure. A positive gate bias of 5 V was selected during irradiation to increase the dosimeter's sensitivity and linearity [29]. Possible signal drifts that occurred in time during and after irradiation have been corrected by using deconvolution methods in this dosimetry system [30]. The stated uncertainty associated with this reader was ±1 mV in the integral mode and was found to be lower than ±2 mV before and after the irradiation in the real-time mode [31].

Prior to use, the MOSFET dosimeter was calibrated against a 0.6 cc ion chamber (TW30013, PTW, German) using the X-ray source from the Elekta XVI system at 120 kV. As adding the filtration would change the X-ray spectrum which may vary the MOSFET sensitivity [22], the calibration was conducted for F0 and F1 filter, respectively. The half value layers (HVL) of the primary X-ray beam at different conditions were determined by using the ion chamber.

During the calibration, the MOSFET dosimeters were placed on the surface of a 40×40×20 cm^3^ solid water slab phantom at the iso-center with their sensitive region facing the x-ray beam. The CBCT scanning parameters used for calibration were listed in Table 6. The delivered dose (D_w,z=0_) was obtained with the ion chamber at the iso-center using the in-air method following the recommendation of AAPM TG-61 report [32]:

$$D_{w,z=0}={\text{MN}}_{K}B_{w}P_{\text{stem},\text{ air}}{\left[{\left(\frac{{\bar{\mu }}_{\text{en}}}{\rho }\right)}_{\text{air}}^{w}\right]}_{\text{air}}$$

where M is the ion chamber reading corrected for temperature, pressure, ion recombination and polarity effect. $N_{K}$ indicates the air-kerma calibration factor relevant to the beam quality. $B_{W}$ indicates the backscatter factor. $P_{\text{stem},\text{ air}}$ indicates the chamber stem correction factor. ${\left[{\left(\frac{{\bar{\mu }}_{\text{en}}}{\rho }\right)}_{\text{air}}^{w}\right]}_{\text{air}}$ indicates the ratio for water-to-air of the mean mass energy absorption coefficients averaged over the incident photon spectrum. The associated calibration parameters were detailed in Table 1.

All the measurements were repeated five times and the average readings were used to calculate the MOSFET sensitivity:

$$\text{Sensitivity}(mV/cGy)=\Delta \text{VTH}(mV)/D_{w,z=0}(cGy)$$ (1)

where ΔVTH is the threshold voltage shift of the MOSFET dosimeter before and after the irradiation.

To evaluate the measurement reproducibility (S), the MOSFETs were scanned with a typical thoracic CBCT protocol (protocol B, see Table 1) using the calibration setup. Measurements were repeated ten times and the reproducibility of the MOSFETs were computed as:

$$S=\frac{1}{\bar{M}}\times \sqrt{\frac{\sum_{i=1}^{n}{\left(\bar{M}-M_{i}\right)}^{2}}{n-1}}\times 100\%$$ (2)

where $\bar{M}$ is the average MOSFET reading for ten equal and consecutive irradiations (n = 10).

### Breast dose measurement

**Figure 1:**
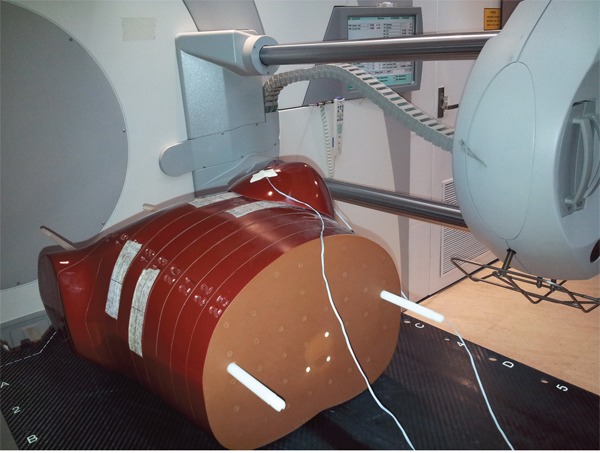
Setup of breast CBCT dose measurement using a female-configured anthropomorphic phantom and MOSFET detectors

As shown in Figure 1, the breast doses were measured with MOSFET dosimeters positioned at 3, 6, 9, 12 o’clock and at the center on breast surface in a female-configured anthropomorphic phantom (The Alderson Radiation Therapy phantom, Radiology Support Devices, Inc. USA). To fully assess the effectiveness of partial arc CBCT scanning, both ipsilateral and contralateral breast were investigated in this experiment. Measurements were performed with artificial breasts of different sizes to determine the influence of breast volume on breast dose. The possible effects of filtration and varying rotation were also tested.

### Analysis of image quality

**Figure 2:**
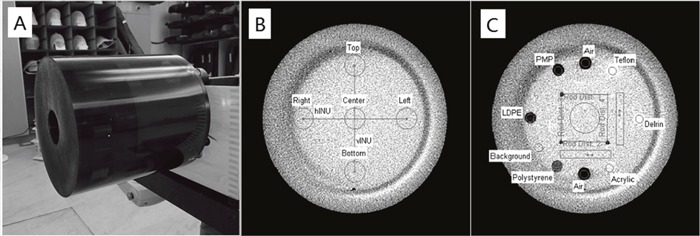
(A) Catphan 503 phantom, (B) images of 486 module used for image uniformity test and (C) 404 module used for low contrast visibility evaluation

The image uniformity and low contrast visibility were investigated in a Catphan 503 phantom (The Phantom Laboratory, Salem, NY, USA) (Figure 2A) to evaluate the impact of dose reduction on CBCT image quality.

### Uniformity

The phantom's homogeneous module CTP 486 was imaged (Figure 2B). Five 1 cm diameter circular regions of interest (ROIs) were selected on the middle slice of the images at the module center and at 3, 6, 9, and 12 o’clock positions. All the peripheral ROIs were carefully set to be within the “inner circle” (uniformity module) of the image, which was 2 cm from the edge of the image area. The mean CT value of each ROI was then determined, by means of which the maximum and minimum CT number was identified. The non-uniformity index was calculated using the formula:

$$\text{Non}-\text{uniformity}=\left(\frac{\text{mean}\left(\text{high}\right)-\text{mean}(\text{low})}{\text{mean}(\text{high})}\right)$$ (3)

### Low contrast visibility

To estimate the low contrast visibility, the module CTP 404 was scanned (Figure 2C). Two circular ROIs with a diameter of 0.35 cm were identified within the low-density polyethylene (LDPE) and polystyrene (PS) inserts, respectively, on the middle layer of the images. The mean pixel values within these ROIs and the associated standard deviations (SD) were recorded. The low contrast visibility was calculated as follows:

$$\text{low contrast visibility}\%=\frac{({\text{CT}}_{\text{polystyrene}}-{\text{CT}}_{\text{LDPE}})/10}{\left\{\frac{\left({\text{mean}}_{\text{polystyrene}}-{\text{mean}}_{\text{LDPE}}\right)}{\left({\text{SD}}_{\text{polystyrene}}-{\text{SD}}_{\text{LDPE}}\right)/2}\right\}}$$ (4)

### Registration accuracy

The female-configured anthropomorphic phantom in Figure 1 was used to analyze the registration accuracy for different CBCT scanning protocols. The phantom was firstly scanned by a spiral CT at 120 kVp with 3 mm slice thickness and 3 mm spacing. The CT images were transferred to the XVI workstation as reference images.

Keeping the same phantom setup, CBCT acquisitions were performed with M20 collimator cassette and F1 filter using varying rotations (i.e., 360° full arc scanning vs. 220° partial arc scanning). The systematic setup and registration error was hence determined by image registration of CT and CBCT images using Elekta Synergy R3.5 software. In this study, the grayscale match algorithm was preferred and the registration box was selected to cover the whole chest area according to the departmental IGRT protocols. The phantom was then shifted by 1 cm in three directions to simulate the patient's inter-fractionation motion. New CBCT images were acquired and the detected positioning error was compared with the nominal shifted distance.
